# Supplementary material for: High-Performance Reversible Furan–Maleimide Resins Based on Furfuryl Glycidyl Ether and Bismaleimides
Source: Polymers (Basel). 2023 Aug 19;15(16):3470. doi: 10.3390/polym15163470 (PMC10459929; doi:10.3390/polym15163470)
Supplement: Supplementary file 1 [file polymers-15-03470-s001.zip › polymers-2502453-supplementary.pdf]

Supplementary Materials

1. Structural characterization of monomers

1.1. Furfuryl glycidyl ether (FGE)

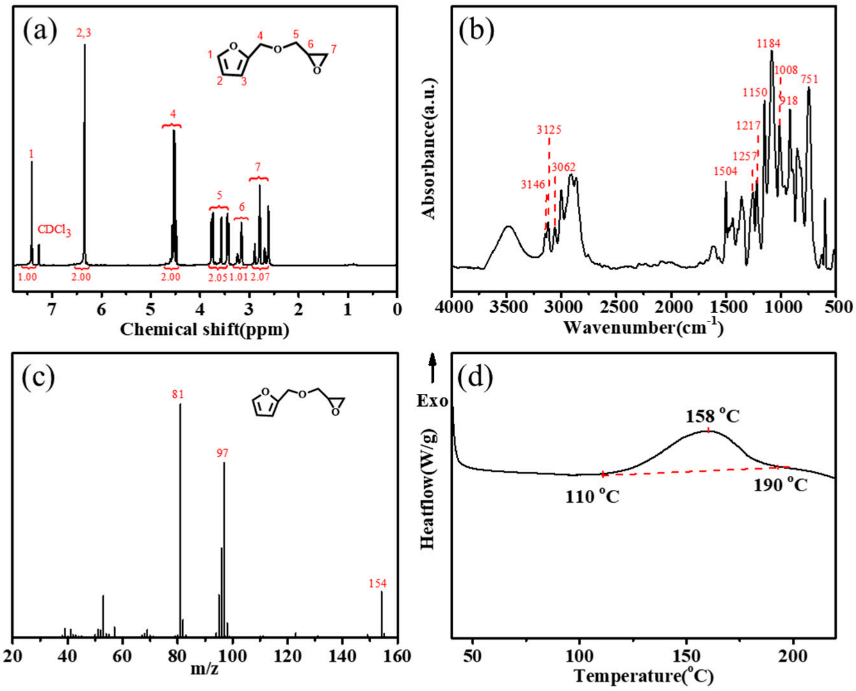

Figure S1. ^{1}H-NMR spectra (a), FT-IR spectra (b), EI-MS spectra (c), and DSC curve (d).

1.2. N, N'-hexamethylene-bismaleimide (HBMI)

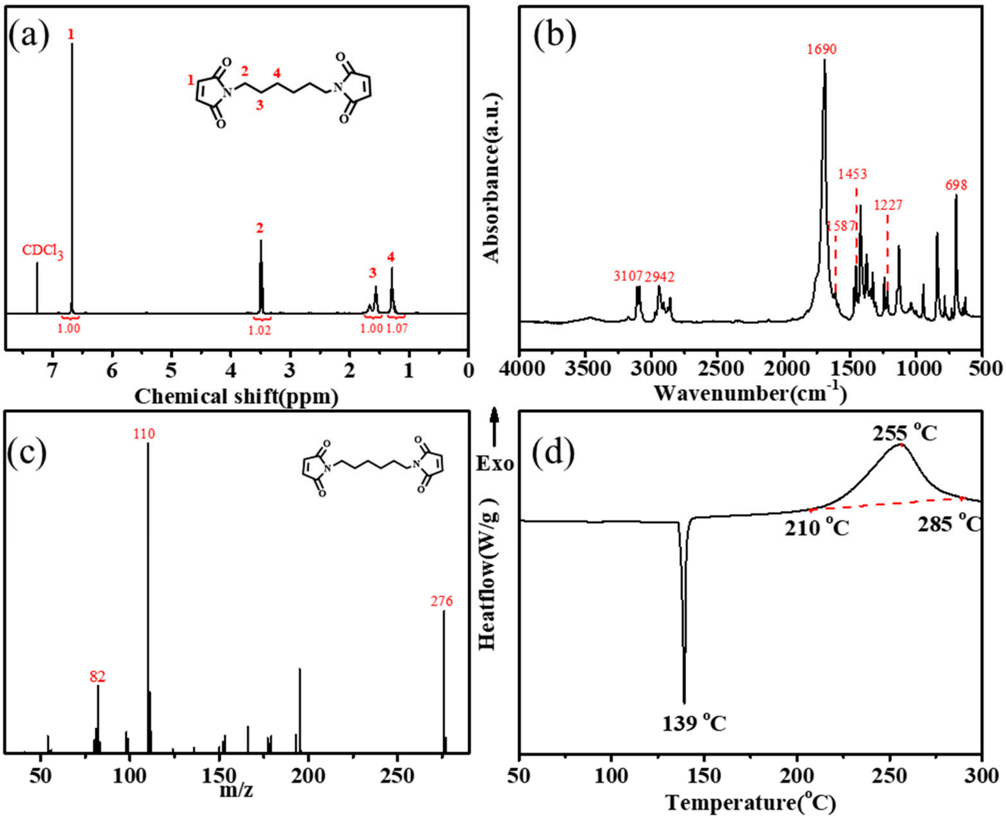

Figure S2. ^{1}H-NMR spectra (a), FT-IR spectra (b), EI-MS spectra (c), and DSC curve (d).

1.3. Investigation of the reaction temperatures of FGE-ODA-BMI resins

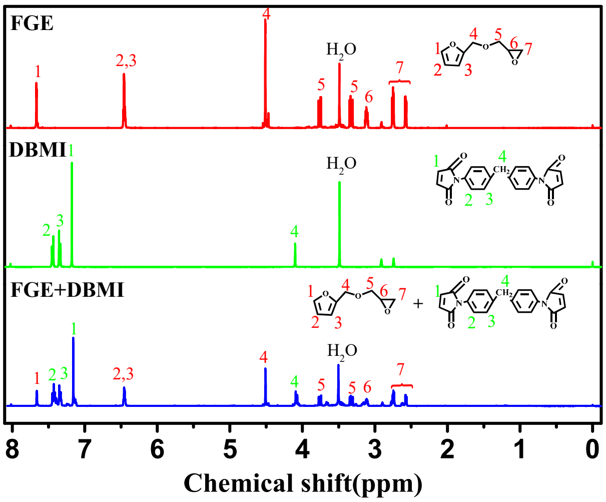

Figure S3. ^{1}H-NMR spectra of FGE, DBMI and FGE+DBMI (the mixture after heating at 55 °C for 30 min).

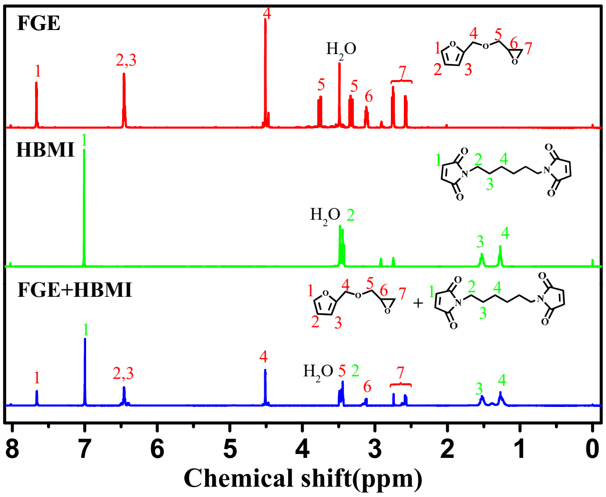

Figure S4. ^{1}H-NMR spectra of FGE, HBMI and FGE+HBMI (the mixture after heating at 55 °C for 30 min).

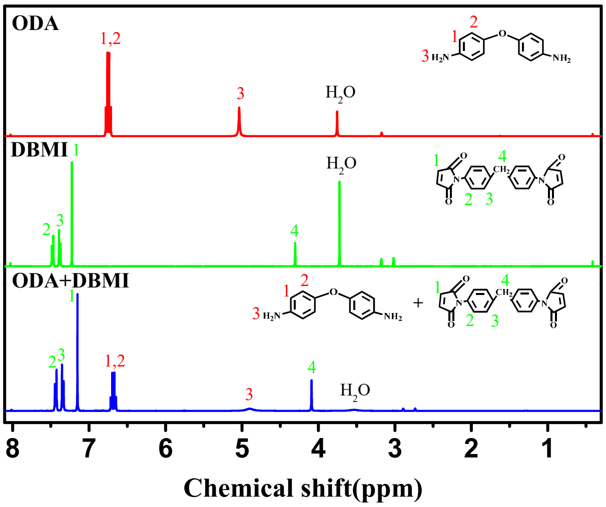

Figure S5. ^{1}H-NMR spectra of ODA, DBMI and ODA+DBMI (the mixture after heating at 125 °C for 30 min).

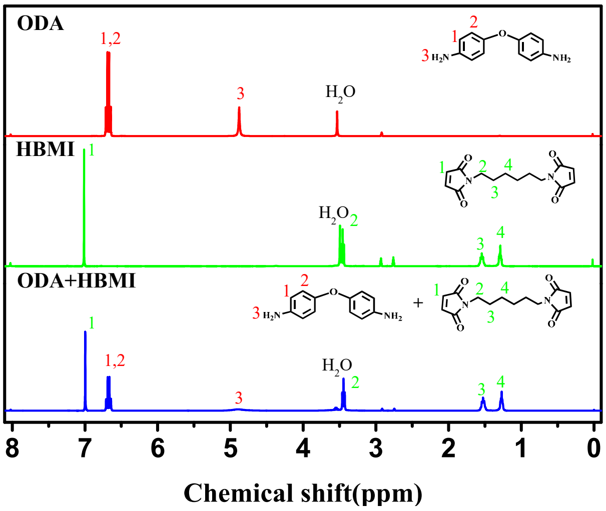

Figure S6. ^{1}H-NMR spectra of ODA, HBMI and ODA+HBMI (the mixture after heating at 125 °C for 30 min).

2. Curing procedures and structural characterization of FGE-ODA-HBMI resin

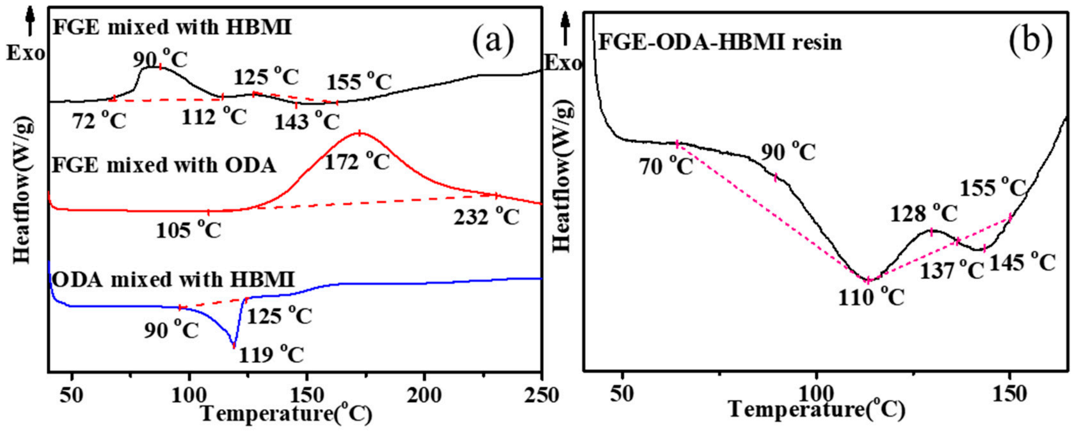

Figure S7. DSC curves of FGE mixed with HBMI, FGE mixed with ODA, ODA mixed with HBMI (a), DSC curves of FGE-ODA-HBMI resin (b).

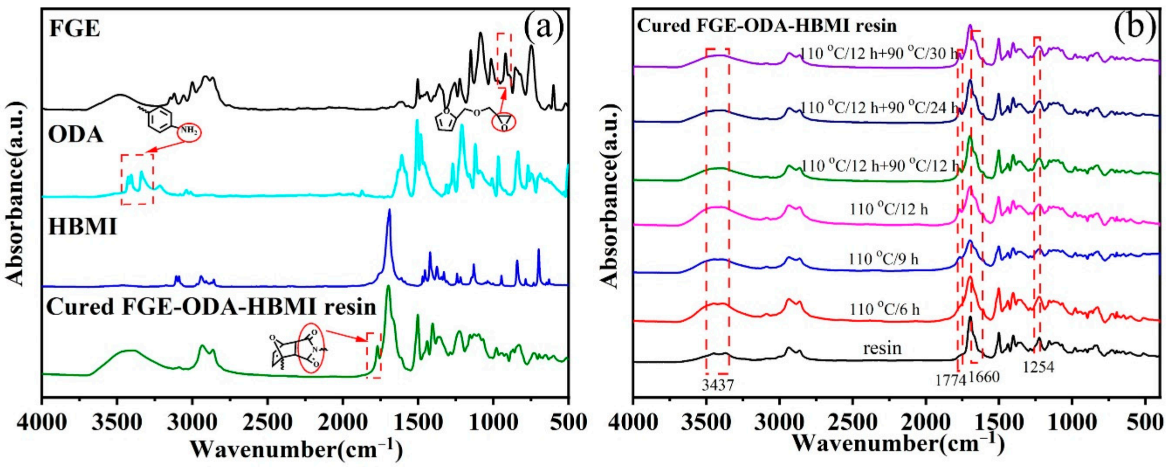

Figure S8. FT-IR spectra of the cured FGE-ODA-HBMI resin and its raw materials(a), and FGE-ODA-HBMI resin at different curing stage (b). The characteristic groups (red circles) are circled and pointed to the characteristic absorption peaks (dashed boxes) in the picture.

3. Reversible performance of the cured FGE-ODA-BMI resins

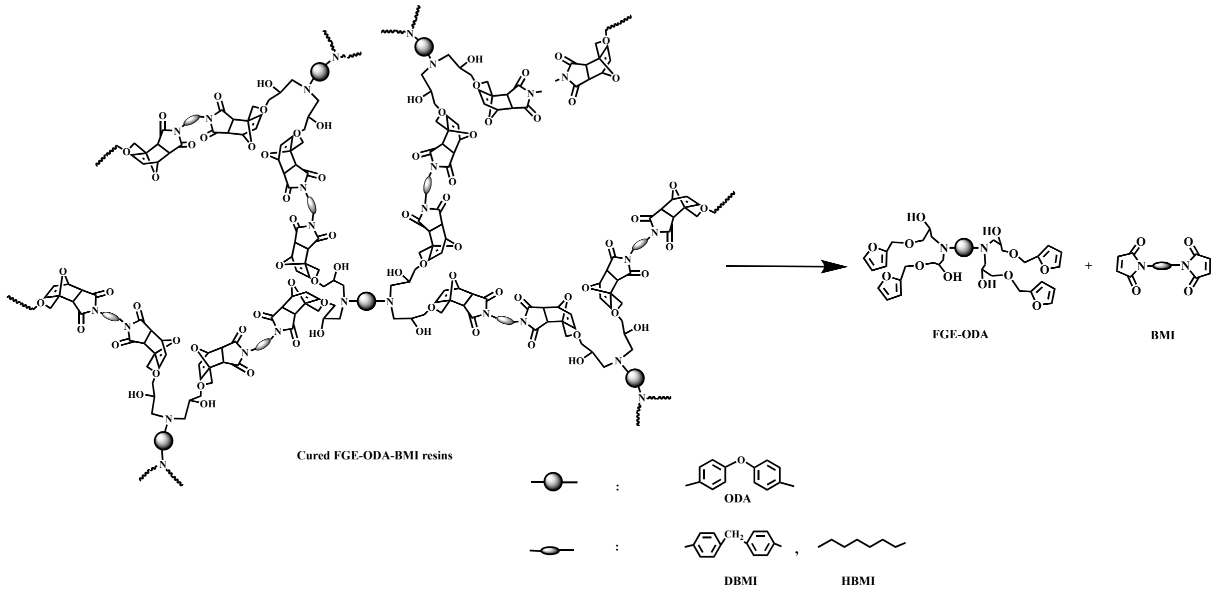

Figure S9**.** Schematic diagram of r-DA reaction of cured resin.

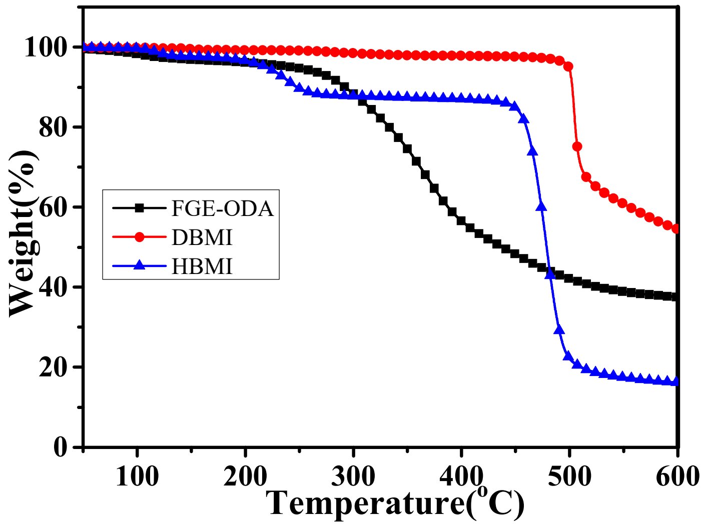

Figure S10. TGA curves of FGE-ODA, DBMI and HBMI.

4. The sol-gel transformation process of the cured FGE-ODA-HBMI resin

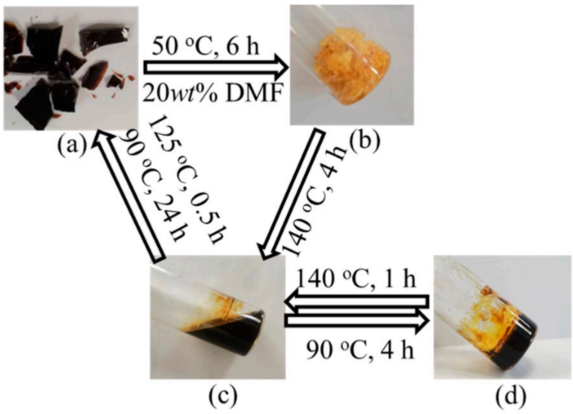

Figure S11. The photos of sol-gel transformation process for the cured FGE-ODA-HBMI resin: the small fragments of cured resin (a), the swelled state of cured resin (b), the dissolved state of cured resin (c), the gel state of cured resin (d).

5. The hot-pressing process of cured resins

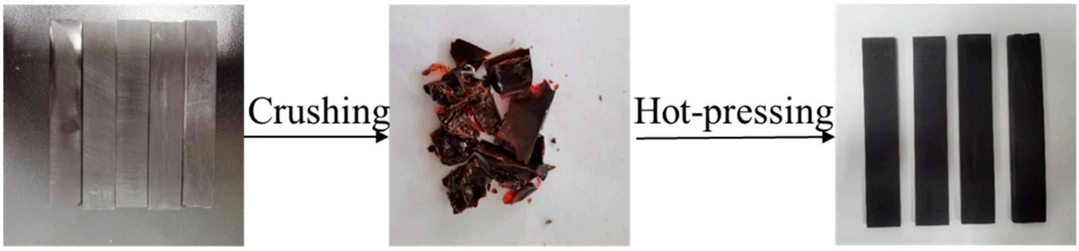

Figure S12. The schematic diagram of the hot-pressing process of cured resins.
